# Magnetic-Transition-Metal Oxides Modified Pollen-Derived Porous Carbon for Enhanced Absorption Performance

**DOI:** 10.3390/ijerph192416740

**Published:** 2022-12-13

**Authors:** Shuyun Tai, Ying Li, Ling Yang, Yue Zhao, Sufei Wang, Jianxin Xia, Hua Li

**Affiliations:** 1 Key Laboratory of Ecology and Environment in Minority Areas, Minzu University of China, National Ethnic Affairs Commission, Beijing 100081, China; 2 College of Life and Environmental Sciences, Minzu University of China, Beijing 100081, China

**Keywords:** transition-metal oxide, biochar, adsorption, recycling

## Abstract

In our work, the transition-metal-oxide precursor (TMO@BC, M = Fe, Co, Ni) has been loaded on the pollen carbon by the hydrothermal method and annealed at different temperatures to generate a composite material of metal oxide and pollen carbon in this study, which can effectively prevent agglomeration caused by a small size and magnetism. The XRD patterns of the samples showed that the as-synthesized metal oxides were γ-Fe_2_O_3_, CoO, and NiO. In the 20 mg/L methyl orange adsorption experiment, the adsorption amount of CoO@C at 500 ℃ reached 19.32 mg/g and the removal rate was 96.61%. Therefore, CoO@C was selected for the adsorption correlation-model-fitting analysis, which was in line with the secondary reaction. The pseudo-second-order kinetic model (*R*^2^: 0.9683–0.9964), the intraparticle diffusion model, and the Freundlich adsorption isotherm model indicated that the adsorption process was the result of both physical and chemical adsorptions, and the judgment was based on the electrostatic action. The adsorption and removal efficiency of ciprofloxacin (CIP) by changing the pH of the reaction was about 80%, so the electrostatic attraction worked, but not the main factor. Recovered by an external magnetic field, the three-time recycling efficiency was still maintained at more than 80%. This novel biomass-derived magnetic porous carbon material embedded with transition-metal-oxide nanoparticles is highly promising for many applications, especially in the field of environmental remediation.

## 1. Introduction

The frequent detection of emerging contaminants (ECs) in wastewater, drinking water, and groundwater has attracted wide attention from the international community as an enormous challenge for water-pollution control [1]. The residual and refractory organics from industrial manufacture and the daily life of humans flow down the sewage system through arbitrary discarding or excretion, which enter into natural water and cause significant detriments through runoff, diffusion, and percolation [2]. The adsorption for pollutant removal from wastewater is a widely and effectively applied method with handleability, nonsecondary pollution, low-cost, and energy consumption [3]. The powder adsorbents that provide a large contact area are mainly suspended in the reaction system, which leads to the cumbersome recovery and exorbitant expenditure; moreover, the way to improve the recycle efficiency of the nanoabsorbent is also one of the key issues in practical applications [4,5].

Transition-metal oxides (TMOs) are a popular category reagent towards contamination control due to their favorable adsorption and diffusion properties, benign environmental compatibility, and excellent chemical and thermal stability [6,7,8,9]. Among these materials, the transition-metal-oxides (TMO) nanocrystals of Fe, Co, and Ni in the fourth row of the periodic table have been extensively studied in the past decades due to their magnetic properties [10,11]. Yu and coworkers [12] synthesized a novel Au-graphene oxide (GO)–Co_3_O_4_ hollow sphere (Au/GO-Co_3_O_4_) and examined its process affected by adsorption in antibiotic-resistance genes (ARGs). Gallo et al. [13] prepared superparamagnetic nanosorbents based on iron oxide by the addition of a porogenic material and calcination for the adsorptive removal of lead and methyl orange from water. A superparamagnetic phenomena of TMOs could be generated by modulating their size to the nanometer, which can diminish the interactions between particles, simultaneously separating and recycling readily with the addition of a low-gradient magnetic field [14].

Research related to carbonaceous adsorbents with a high specific surface area and abundant pore structures has increased in the past decades, such as activated carbon, graphite, charcoal and rice char, etc. [15,16] Niu et al. fabricated the CoO/g-C_3_N_4_ by an impregnation–calcination method for tetracycline (TC) removal, which exhibited a high-adsorption capacity and fast-adsorption kinetic for TC [17]. Esra et al. [18] synthesized a magnetic activated carbon (AC) (Fe-AC) for the effective removal of Methylene Blue (MB) by means of adding the iron oxide to the AC obtained by the ZnCl_2_ activation of acorn shell. Biochars are carbon-rich materials made from heating biomass in a little-or-no-oxygen atmosphere at relatively low temperatures (<700 °C), which is regarded as an environmentally friendly adsorbent for pollutant removals such as oxytetracycline, polycyclic aromatic hydrocarbon, and polychlorinated biphenyls [19,20,21,22,23,24,25,26,27]. Magnetic biochars could be formed by introducing transition-metal oxides (Fe, Co, Ni) into biochar matrices by the hydrothermal method, the sol-gel method, the dielectrophoresis method, and so on, which are effective strategies to recycle the powder and avoid the magnetic agglomeration of nanometal oxides [28,29,30,31].

In this study, a widely sourced green biomass carbon made from pollen was used as the carrier of transition-metal-oxide (Fe, Co, Ni) nanoparticles, and the network of hollow skeletons after carbonization was fully utilized to provide a larger contact area for Fe_2_O_3_/CoO/NiO to prevent the agglomeration of nanoparticles. The results indicate that the composite prepared by hydrothermal method has a large specific surface area; meanwhile, the biochar interacts with the transition-metal oxides, which exhibits the much enhanced adsorption performance of Fe_2_O_3_/CoO/NiO@C. The paramagnetic and ferromagnetic manifestation of Fe_2_O_3_/CoO/NiO@C materials still appear after loading pollen carbon, which can be separated under the condition of an external magnetic field, recycled, and maintain a superior removal rate after the reaction.

## 2. Experimental Section

### 2.1. Chemicals

Fe(NO_3_)_3_·9H_2_O, Co(NO_3_)_2_·6H_2_O, and Ni(NO_3_)_2_·6H_2_O (99.5%) were obtained from Sigma-Aldrich (China). Methanol, NaOH, HCl, anhydrous ethanol (99.7%), ethylene glycol, methyl orange (MO), and sodium citrate were purchased from Sinopharm Chemical Reagent Co., Ltd. (Beijing, China). Norfloxacin (NOR) and ciprofloxacin (CIP) were provided by Solarbio (China). Rape pollen were purchased from Qinghai, China. All of the chemicals were of analytical grade and used without further purification. Deionized water was used for all synthesis and treatment processes. Deionized water (resistivity > 18.2 MΩ cm/25 °C) was obtained from a Millipore water system (Millipore Corp. Bedford, MA, USA) at room temperature.

### 2.2. Preparation of Fe_2_O_3_/CoO/NiO@C Composites

Carbonized pollen were prepared by the following processing: 2.5 g rape pollen were added into a beaker and mixed with 30 mL distilled water, treated for approximately 10 min in an ultrasonic bath, then washed completely with 30 mL of absolute ethanol with the above step and placed in an 80 °C oven to dry. The dried pollen were added into mixed with 40 mL distilled water. The mixture solution was ultrasonicated before being transferred into a 50 mL Teflon-lined autoclave. The autoclave was then heated and kept in an oven at 180 °C for 24 h. The obtained brown–black precipitates were washed by deionized water and ethanol and then dried at 60 °C overnight in a vacuum.

**Figure 1:**
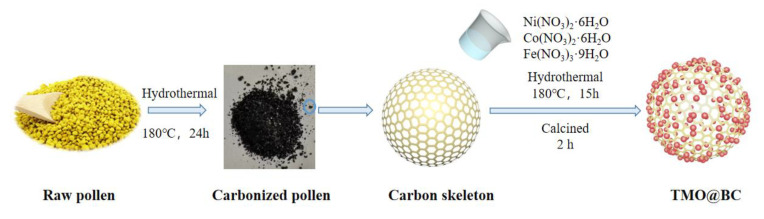
Schematic diagram of the hydrothermal method for Fe_2_O_3_/CoO/NiO@C.

Fe_2_O_3_/CoO/NiO@C were prepared by the following process: 0.1 g carbonized pollen, 2 mmol transition metal nitrate, and 1 mmol sodium citrate were added into the mixture of 40 mL ethylene glycol and distilled water (1:1), then prepared under vigorously continuous magnetic stirring for 30 min at an indoor temperature, and ultrasonicated for 15 min in the ultrasonicator. The mixture solution was transferred into an autoclave, then heated and kept in an oven at 180 °C for 15 h. The precipitates were washed with deionized water and ethanol and then dried at 60 ℃ in an oven. The dried sample was taken out and put into a tube furnace, then annealed from room temperature to calcination temperature of 400, 450, and 500 °C at a heating rate of 5 °C/min in the N_2_ flow for 2 h, termed as the Fe_2_O_3_/CoO/NiO@C-400, Fe_2_O_3_/CoO/NiO@C-450, and Fe_2_O_3_/CoO/NiO@C-500, respectively. The schematic diagram of hydrothermal method for Fe_2_O_3_/CoO/NiO@C is presented in Figure 1.

### 2.3. Characterization Methods

The surface morphology of the composites were characterized by scanning electron microscopy (SEM, Hitachis-4800). The crystallinity of the materials were investigated using the XD-3 X-ray diffractometer with a Cu target (Kα radiation, λ ¼ 1.5406 Å) from 20° to 80°. Fourier transform infrared (FT-IR) spectra of the products were collected using a Vertex 70 v with KBr pellets. The Brunauer–Emmett–Teller (BET) surface areas of all prepared photocatalysts and N_2_ adsorption–desorption isotherm data were carried out by the ASAP 2460 Surface Area and Porosity Analyzer.

### 2.4. Exploration of the Performance of Adsorption

The adsorption performance of the Fe_2_O_3_/CoO/NiO@C powders was evaluated by the removal of MO. Several parameters have been changed individually for further exploration. An amount of 50 mg of the samples at different calcination temperatures were used to adsorb the MO/CIP/NOR solution (20 mg/L, 50 mL) in a dark condition, respectively. Approximately 4 mL of aqueous solution was collected at certain time intervals, centrifuged at a speed of 5000 r/min for 15 min, and filtered with a 0.22 µm syringe filter. The concentration of samples was monitored by a UV–vis spectrophotometer (TU-1901) at their characteristic wavelengths (λ_MO_ = 464 nm, λ_CIP,_ λ_NOR_ = 272 nm). The degradation rate was calculated by Equation (1):

(1) $$\mathrm{Removal}\;\mathrm{rate}\;(\%)=\frac{\left(C_{0}-C_{t}\right)}{C_{0}}\times 100\%$$

where *C*_0_ (mg/L) and *C_t_* (mg/L) are the initial and at time t concentrations of the pollutant, respectively. As the most important data related to adsorption kinetic and equilibrium studies, the corresponding adsorption capacity was computed by Equation (2) [28,29]:

(2) $$q_{t}=\frac{(C_{0}-C_{t})V}{m}$$

where *V* (L) is the initial volume of pollutant solution and *m* (g) denotes the mass of the adsorbent.

### 2.5. Kinetic Models and Adsorption Isotherms

The kinetic adsorption experiments were performed by adding 50 mg of the CoO@C-500 into the 20, 50, and 80 mg/L MO concentration solutions at 298.15 K. The initial MO concentrations of the solution were in the range of 10–100 mg/L in the isotherm adsorption experiments. In order to investigate the adsorption behavior of the synthesized CoO@C-500, the pseudo-first order, pseudo-second order, Elovich kinetic models, and intraparticle diffusion model were fitted by the experiment data, as well as the Langmuir and Freundlich isotherm models to further assure the style of adsorption. 

The adsorption rate of the pseudo-first-order kinetic is proportional to the concentration change value. The linear form of the pseudo-first-order kinetic model was calculated by Equation (3) [32]:

(3) $$\ln(q_{e}-q_{t})=\ln q_{e}-k_{1}t$$

The adsorption process is controlled by chemical mechanisms, including the electrons shared and transfer between adsorbents and adsorbates in the pseudo-second-order kinetic model. The linear form of the pseudo-second-order kinetic model was given by Equation (4) [33]:

(4) $$\frac{t}{q_{t}}=\frac{1}{k_{2}{q_{e}}^{2}}+\frac{t}{q_{e}}$$

where *q_e_* (mg/g) and *q_t_* (mg/g) are the adsorption capacity at equilibrium at time *t* (min), respectively, and *k*_1_ (min^−1^) and *k*_2_ (g/mg·min^−1^) are the pseudo-first-order and pseudo-second-order rate constants, respectively.

The Elovich kinetic equation is one of the heterogeneous diffusion models describing the chemisorption process, defined as Equation (5) [32]:

(5) $$q_{t}=\frac{1}{b}\ln(ab)+\frac{1}{b}\ln t$$

where *a* (mg/g·h) is the initial sorption rate, and the value of ($\frac{1}{b}$) is indicative of the available number of sites for adsorption.

The intraparticle diffusion model is controlled by internal diffusion and external diffusion. The linear form of the intraparticle diffusion model is calculated by Equation (6) [34]:

(6) $$q_{t}=k_{i}t^{1/2}+C$$

where *K_i_* (mg/g·min^1/2^) is the intraparticle diffusion rate constant and *C* is the boundary layer thickness.

The isotherm adsorption experiments were used to explore the mechanism of adsorption by analyzing the relationship between the equilibrium adsorption concentration and the equilibrium adsorption capacity. The Langmuir and Freundlich adsorption isothermal equation (Formulas (7) and (8)) were used to fit the experimental data [35].

(7) $$q_{e}=\frac{k_{L}q_{m}C_{e}}{1+k_{L}C_{e}}$$

(8) $$q_{e}=k_{F}{C_{e}}^{1/n}$$

where *Ce* (mg/L) is the equilibrium concentration of MO, *q_e_* (mg/g) is the adsorption capacity at equilibrium, and *q*_m_ (mg/g) is the maximum monolayer adsorption capacity. *K_L_* (L/mg) is the Langmuir model constants, *K_F_* [mg·g ^−1^·(L·mg^−1^)^1/n^] is the Freundlich model adsorption coefficient, and 1/*n* is the adsorbent surface heterogeneity index.

### 2.6. Recycled Application of CoO@C-500

In this study, 50 mg of the adsorbent was taken in 50 mL MO with initial concentration of 20 mg/L and stirred for 150 min to attain equilibrium. Then, the adsorbent was washed with distilled water, and the desorption of the adsorbed molecules was carried out using methanol. The adsorption–desorption process was repeated for three cycles.

## 3. Results and Discussion

### 3.1. Morphologic Features and Structural Analysis

**Figure 2:**
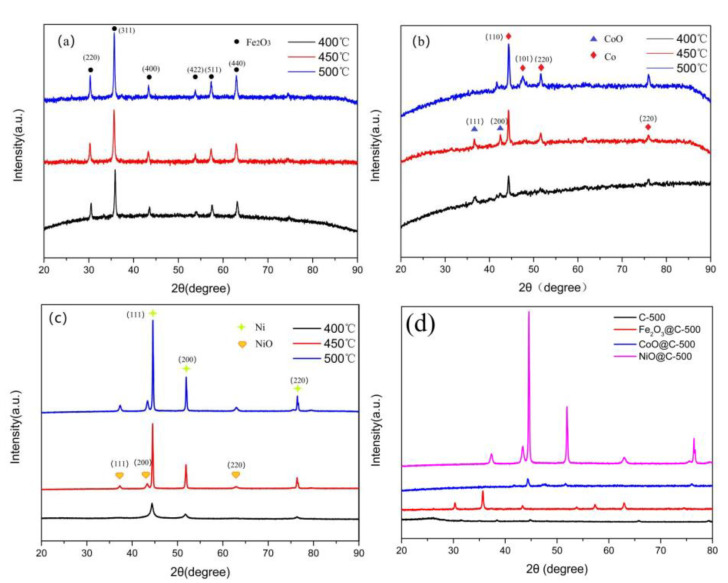
XRD patterns of Fe_2_O_3_@C (**a**), CoO@C (**b**), and NiO@C (**c**) at different calcination temperatures, and the Fe_2_O_3_/CoO/NiO@C-500 (**d**).

The crystal structures of the samples obtained at different annealing temperatures were characterized by XRD, and the results are shown in Figure 2. The XRD patterns of Fe_2_O_3_, CoO, NiO, and the pollen carbon at different calcination temperatures are shown in Figure S1. The spectral curve fluctuated more than the pure metal oxides due to the intervention of doped-amorphous carbon. The composition of the crystal changed obviously with the increasing of the annealing temperature from 400 °C to 500 °C. As illustrated in Figure 2a, the XRD patterns of the samples showed that the as-synthesized metal oxides were γ-Fe_2_O_3_. The Fe_2_O_3_@C showed diffraction peaks at 30.2° (220), 35.6° (311), 43.3° (400), 53.7° (422), 57.3° (511), and 62.9° (440). As displayed in Figure 2b, the CoO@C crystal plane diffraction 2θ angle corresponded to 37.8° and 42.5°, according to the standard card of PDF#43-1004, which indexed to the (111) and (200) crystal faces of the CoO nanoparticles, respectively. Figure 2c exhibited the XRD patterns of the as-prepared NiO@C samples, where the diffraction peaks corresponded to the (111), (200), and (220) lattice planes of NiO, respectively (JCPDS no. 47-1049). In addition, part of the CoO and NiO was converted into Co and Ni nanoparticles on account of the synergistic reduction of carbon and hydrogen during the annealing. The diffraction peaks were more intense and narrower at 500 °C, implying the high crystallinity of Fe_2_O_3_@C, CoO@C, and NiO@C, as shown in Figure 2d. Therefore, the samples calcined at 500 ℃ were selected for the detailed characterization.

**Figure 3:**
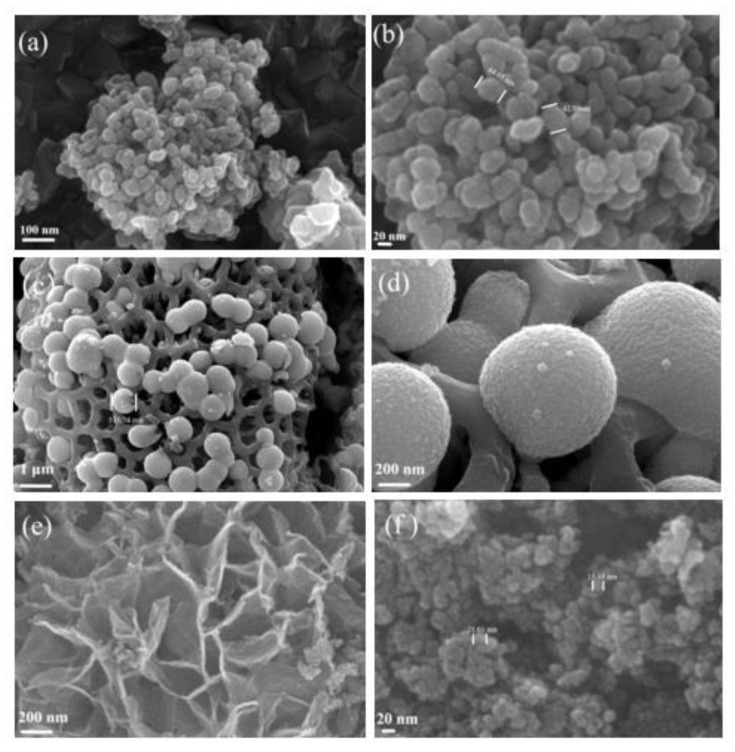
SEM images of Fe_2_O_3_@C-500 (**a**,**b**), CoO@C-500 (**c**,**d**), and NiO@C-500 (**e**,**f**).

The microstructures of the Fe_2_O_3_@C, CoO@C, and NiO@C samples were characterized by SEM (Figure 3). As shown in Figure 3a,b, the Fe_2_O_3_ sample possessed a particle size around 40 nm with the aggregation, which mainly grew microspheres and clusters formed by the accumulation of dense microspheres. As marked in Figure 3c,d, the CoOs were microsphere-type nanostructures and part of the samples were dumb-bell-shaped after the combination of the two microspheres. The microspheres were self-assembled by smaller spherical particles, and the average size of the large sphere was about 400 nm and the size of the nanosphere ranged from 5 to 10 nm. The reticulated surface of the pollen particles were adhered by a large number of CoO and embedded in the carbon skeleton, which could provide more adsorption sites to enhance the adsorption capacity. The SEM images for the NiO in Figure 3e,f show clearly flake clusters and microsphere morphologies. It can also be seen from Figure 3e that there were some nanospherical particles of about 20 nm at the edge of the cluster morphology.

The specific surface was applied to characterize the performance, surface state, and pore structure of the composites sample. The N_2_ adsorption/desorption isotherms and corresponding BJH (Barrett–Joyner–Halenda) pore-size-distribution curves of the Fe_2_O_3_@C-500, CoO@C-500, and NiO@C-500 samples are shown in Figure S2. As displayed in Figure S2a,c, the adsorption isotherm of the Fe_2_O_3_/NiO@C-500 conformed to the V-shaped isotherm, which was concave. The adsorption layers that reached saturated vapor pressure were limited, and the capillary coagulation led to a rapid rise in the medium-pressure part, accompanied by a hysteresis loop. The N_2_ adsorption/desorption isotherms of CoO@C-500 are shown in Figure S2b, which reveal that the isotherms with a distinct hysteresis loop at a lower-relative-pressure (0.4 < P/P_0_ < 1) range could be identified as the typical type IV curve with H_2_-type according to the definition of the IUPAC classification and the implied the existence of mesopores. It could be seen that the D_p_ of Fe_2_O_3_/CoO/NiO@C-500 was 20, 4.3, and 3.9 nm, as shown in Table 1, beyond that the specific surface area of Fe_2_O_3_@C-500, CoO@C-500, and NiO@C-500, which was 44.28, 118.44 and 184.20 m^2^/g, respectively.

### 3.2. Adsorption Properties 

**Figure 4:**
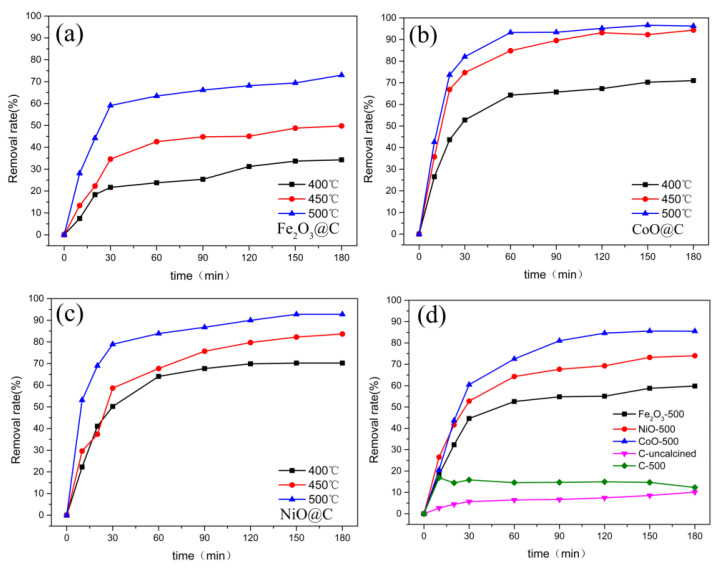
Removal efficiency of MO in the adsorption process on Fe_2_O_3_@C (**a**), CoO@C (**b**), NiO@C (**c**), pure metal oxides, and pollen carbon (**d**).

The adsorption performances of Fe_2_O_3_/CoO/NiO@C were evaluated by monitoring the degradation of the MO in the solution (Figure 4). The adsorptive removal of the MO increased from 34.28% to 72.97% on Fe_2_O_3_@C with the increasing of the annealing temperature from 400 to 500 °C, as shown in Figure 4a. However, the adsorption efficiency was limited by its small specific surface area. As illustrated in Figure 4b, the removal rate of 71.03%, 94.37%, and 96.26% was absorbed by CoO@C at 400 °C, 450 °C, and 500 °C, respectively. Actually, the adsorption equilibrium has been basically reached after 60 min of reaction. The adsorption removal curves of 20 mg/L MO on NiO@C is shown in Figure 4c. The maximum adsorption efficiency on NiO@C ranged from 70.24% to 92.76% with annealing temperature increases owing to the large specific surface area and the good adsorption capacity.

The hemicellulose and cellulose in the biomass were pyrolyzed to form pores as the temperature increases, which in turn affected the adsorption effect [36]. It was found that the much-enhanced adsorption effects of Fe_2_O_3_/CoO/NiO@C-500 more than the pure transition-metal oxides and pollen carbon, as shown in Figure 4d. The adsorption rate of the MO on the pure Fe_2_O_3_/CoO/NiO-500, uncalcined pollen carbon, and C-500 was 59.79%, 85.5%, 74.02%, 10.11%, and 16.86%, respectively. The specific surface area and adsorption sites of the adsorbents increased due to the addition of the pollen carbon skeleton. Meanwhile, the reduction of some metal oxides into metal elements at high temperatures also improved the removal capacity of the pollutant.

**Figure 5:**
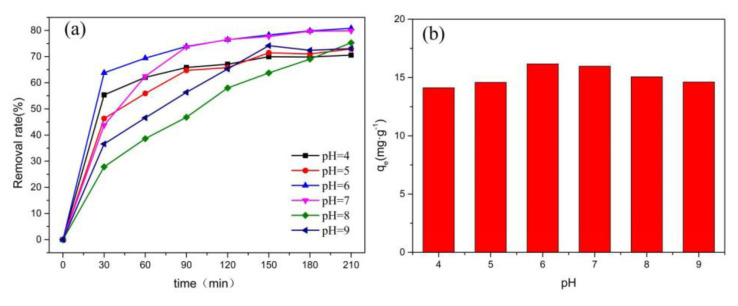
Adsorption rate of ciprofloxacin on CoO@C-500 at different pH conditions (**a**) and equilibrium adsorption capacities (**b**).

The initial pH of the solution could affect the degree of dissociation of the chemical functional groups on the surface of the adsorbent [37]. The structures of the pollutant and the pollen carbon would be destroyed in strong acid and alkali environments, and CoO@C-500 was used to adsorb 20 mg/L ciprofloxacin (CIP) and control the pH of the solution at the range of 4–9 by HCl and NaOH. Antibiotics with complex structures were selected to explore the influence of pH because methyl orange is sensitive to ambient pH as a common acid-base indicator. The maximum removal rate of the CIP on CoO@C-500 was 80.83% and the equilibrium adsorption amount was 16.17 mg·g^−1^ when the pH value was 6.0, as shown in Figure 5. A great deal of investigations have reported the percentage removal of contaminants increased as a function of the adsorbent dosage before reaching a saturation value, which is often attributed to the abundant availability of the vacant sites at higher dosages [38,39,40]. Norfloxacin (NOR) is the same quinolone antibiotic as CIP and is widely used to deal with bacterial diseases in organisms. The effect of the adsorbent dosage of Fe_2_O_3_/CoO/NiO@C-500 for the degradation of 20 mg/L of NOR is shown in Figure S3. The results show that 15.07%, 27.18%, and 51.89% of NOR are absorbed by 25 mg Fe_2_O_3_@C-500, CoO@C-500, and NiO@C-500 for 100 min, respectively, whereas 32.01%, 57.01%, and 86.54% of NOR are absorbed by 50 mg Fe_2_O_3_/CoO/NiO@C-500 with the same conditions.

### 3.3. Adsorption Kinetics and Equilibrium Studies

**Figure 6:**
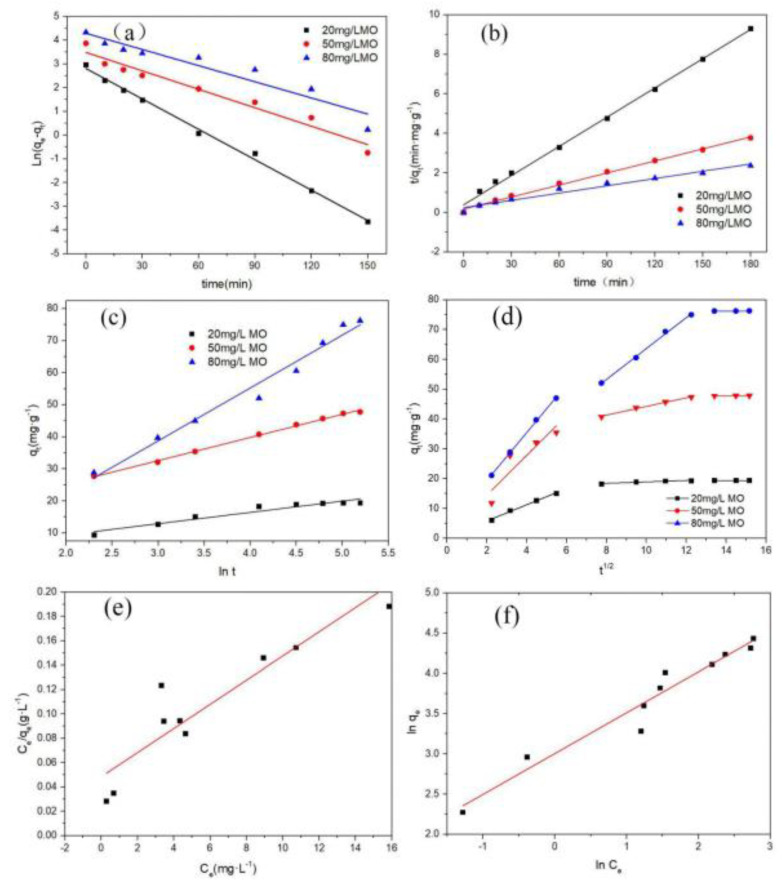
Pseudo−first−order model (**a**), pseudo−second−order model (**b**), Elovich model (**c**), and Intraparticle diffusion model (**d**) of MO on CoO@C-500; Langmuir (**e**) and Freundlich (**f**) model fitted adsorption isotherms of CoO@C-500.

CoO@C annealed at 500 °C was selected for the adsorption correlation model fitting analysis because of its favorable reaction and removal rate. The experimental data were applied for simulating the four models of pseudo-first-order and pseudo-second-order kinetic models, the Elovich model, and the intraparticle diffusion model to explore the effect of the initial pollutant concentration. The fitting results of the pseudo-first-order and pseudo-second-order kinetic models are shown in Figure 6a–c. The slope, correlation coefficient, and equilibrium adsorption capacities are shown in Table 2 and Table 3. It can be seen that the pseudo-second-order model with the correlation coefficient of 0.9683–0.9964 is more suitable for the experimental data by contrast with the pseudo-first-order model (*R*^2^: 0.8961–0.9957) and the Elovich model (*R*^2^: 0.9231–0.9971). The pseudo-second-order equation contains adsorption processes such as membrane diffusion, particle diffusion, and surface adsorption, and it is not a single-diffusion model [41]. The pseudo-secondary model controls the adsorption process based on chemical mechanisms, including electron sharing and electron transfer, with the adsorbate and the adsorbent [42].

The intraparticle diffusion equation was used to fit dynamic data for the in-depth analysis of the diffusion mechanism and practical control steps on CoO@C-500. As shown in Figure 6d and Table 4, the higher k_d1_ reflected on the surface diffuses faster and the adsorption rate is larger, and the linear segment did not go through the origin, demonstrating that the adsorption process is controlled by the surface adsorption along with the pore diffusion. For the second linear segment, the *k_d_*_2_ decrease might be due to the increase of the boundary-layer effect and mass-transfer resistance on the intraparticle diffusion process. At the third stage, the solid–liquid phase distribution was gradually balanced and the adsorption process reached equilibrium, which led to the further reduction of k_d3_ [43,44,45].

The results fitted with the Langmuir and Freundlich isothermal equations are shown in Figure 6e,f and Table 5. The MO solution with concentration range of 10–100 mg/L was used as the target pollutant at 298 K, then 50 mg of adsorbent was added and stirred for 180 min. The Freundlich isothermal equations (*R*^2^: 0.9462) are more suitable for analyzing the isothermal adsorption behavior than the Langmuir isothermal equations (*R*^2^: 0.8796, RL: 0.0107–0.09731) according to the above fitting data, which indicates the uniform surface adsorption and the suitability for physical and chemical adsorption.

### 3.4. Magnetism and Stability

**Figure 7:**
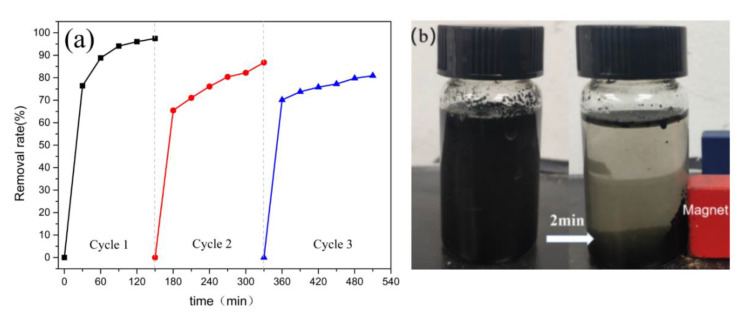
Reusability test for adsorption of MO by CoO@C-500 (**a**); Schematic of the magnetic separation (**b**).

The reusability and regeneration of the adsorbent is an important economical factor for practical utility. We reused the CoO@C-500 after consecutive adsorption experiments to study the regeneration of the adsorbent. As shown in Figure 7a, the adsorption efficiency of CoO@C remained almost above 80% after three cycles, which indicates that the CoO@C exhibited high stability and reusability. Figure 7b shows that CoO@C-500 rapidly gathered near the magnet within 2 min under the condition of the applied magnetic field, indicating that this material can be recycled easily.

### 3.5. Possible Mechanism Analysis

According to the result from pseudo-second-order kinetic models, internal diffusion model, and Freundlich isothermal equations, CIP adsorption by CoO@C-500 involved both physisorption and chemisorption. On the basis of the N_2_ adsorption–desorption result, the specific surface area of CoO@C-500 is 118.44 m^2^/g, which could provide adsorption sites for uptaking the CIP. The adsorption process is in connection with the electrostatic attraction, π bond accumulation, hydrogen bond formation, and hydrophobic interaction generally [46]. The adsorption of MO by CoO@C-500 is not only connected with the mesoporous adsorption in the adsorbent, but also related to the electrostatic interaction between the adsorbent and the surface charge of the adsorbent. The MO usually exists in the form of sulfate with a substantial negative charge in an aqueous solution, and the large amount of positive charge around CoO causes the electrostatic attraction of MO [47]. The FT-IR spectra of CoO@C at the wavenumber between 400 and 4000 cm^−1^ is shown in Figure S5. The absorption peak at 3415 cm^−1^ might be the characteristic vibration of the -OH group. The region between 2924 and 2854 cm^−1^ was related to the C-H groups and the peaks at 1642 and 519 cm^−1^ belong to the C=C and Co-O groups. The influence of pH on CoO@C adsorption of CIP was inconspicuous in the process of exploring the influence of pH on CIP adsorption, indicating that electrostatic attraction was not the dominant factor. The main driving forces for adsorption are the hydrogen bonds formed and the interactions of the π–π bond between the pollen carbon and hydroxyl groups on the CIP and NOR surface.

## 4. Conclusions

A novel adsorbent of Fe_2_O_3_/CoO/NiO@C was successfully fabricated via a solvothermal method and applied to the removal of MO/CIP/NOR. The characterization results showed that the hollowed-out pollen carbon framework was covered with the transition-metal-oxide nanoparticles. The large surface area and high pore volume of metal oxides provide more active sites for the adsorption. Several factors affecting the adsorption were explored and which produced a favorable adsorption capacity. A faintly acidic condition was considered as the best pH condition for the CIP adsorption. The pseudo-second-order model, intraparticle diffusion model, and Freundlich adsorption isotherms model have proved that the adsorption is controlled by both physical and chemical mechanisms. It is effortless and timesaving to separate the adsorbent with an external magnetic field by taking advantage of the magnetism of Fe_2_O_3_/CoO/NiO@C.

## Figures and Tables

**Table 1:** Pore-structure characteristics of adsorbents.

Samples	D_p_ (nm)	S_BET_ (m^2^/g)
Fe_2_O_3_@C	20	44.28
CoO@C	4.3	118.44
NiO@C	3.9	184.20

**Table 2:** Kinetic parameters for the adsorption of MO on CoO@C-500.

*C*_0_ (mg/L)	*q_e_*_.,_ *_exp_* (mg·g^−1^)	Pseudo-First-Order			Pseudo-Second-Order		
		*k*_1_ (1/min)	*q_e_*_,_ *_calc_* (mg·g^−1^)	*R* ^2^	*k*_2_ (g·mg^−1^)	*q_e_*_,_ *_calc_* (mg·g^−1^)	*R* ^2^
20	19.3486	0.0426	16.5008	0.9957	0.0492	19.4767	0.9964
50	43.0392	0.0259	29.7807	0.9547	0.0202	47.2763	0.9957
80	58.5185	0.0226	52.3151	0.8961	0.0122	61.4439	0.9683

**Table 3:** Elovich model parameters of MO on CoO@C-500.

*C*_0_ (mg/L)	*q_e_*_., *exp*_ (mg·g^−1^)	Elovich		
		*k*_3_	*q_e_*_, *calc*_ (mg·g^−1^)	*R* ^2^
20	19.3486	3.5277	20.5962	0.9231
50	43.0392	7.2263	48.4091	0.9971
80	58.5185	16.5083	63.4468	0.9738

**Table 4:** Intraparticle diffusion model parameters of MO on CoO@C-500.

*C*_0_ (mg/L)	*k_d_*_1_ (mg/g·min^1/2^)	*k_d_*_2_ (mg/g·min^1/2^)	*k_d_*_3_ (mg/g·min^1/2^)	(*R*_1_)^2^	(*R*_2_)^2^	(*R*_3_)^2^
20	2.71989	0.24046	0.01676	0.98909	0.89561	0.98587
50	6.69038	1.44694	0.01394	0.75175	0.98824	0.95756
80	8.01921	5.18212	0.01124	0.99853	0.99605	0.96564

**Table 5:** Adsorption isotherms parameters of MO on CoO@C-500.

*T* (K)	*q_e_*_., *exp*_ (mg·g^−1^)	Langmuir				Freundlich		
		*K_L_*	*R_L_*	*q_m_* (mg·g^−1^)	*R* ^2^	*K_F_*	*n*	*R* ^2^
298	19.3486	0.9246	0.0107–0.09731	23.46	0.8796	20.2468	1.9724	0.94618

## Data Availability

Not applicable.

## Supplementary Materials

The following supporting information can be downloaded at: https://www.mdpi.com/article/10.3390/ijerph192416740/s1, Figure S1. XRD patterns of Fe2O3 (a), CoO (b), NiO (c) and pollen carbon (d) at different calcination temperatures; Figure S2. Adsorption isotherms and differential pore size distributions in a semi-logarithmic scale of Fe2O3@C-500 (a), CoO@C-500 (b) and NiO@C-500 (c); Figure S3. Adsorption degradation of Norfloxacin with 25 mg and 50 mg Fe2O3@C-500 (a), CoO@C-500 (b) and NiO@C-500 (c); Figure S4. Removal efficiency of 20 mg/L MO (a) and CIP/NOR (b) in the adsorption process on pollen carbon (a); Figure S5. FT-IR of C-500 and CoO@C-500 (a), FT-IR of the MO (b), CIP (c) and NOR (d) before and after adsorption by carbon.
