# Supplementary material for: Magnetic-Transition-Metal Oxides Modified Pollen-Derived Porous Carbon for Enhanced Absorption Performance
Source: Int J Environ Res Public Health. 2022 Dec 13;19(24):16740. doi: 10.3390/ijerph192416740 (PMC9778859; doi:10.3390/ijerph192416740)
Supplement: Supplementary file 1 [file ijerph-19-16740-s001.zip › ijerph-2033473-supplementary.pdf]

Magnetic-Transition-Metal Oxides Modified Pollen-Derived Porous Carbon for Enhanced Absorption Performance

Shuyun Tai [1,2], Ying Li [1,2], Ling Yang [1,2], Yue Zhao [1,2], Sufei Wang [1,2], Jianxin Xia [1,2] and Hua Li [1,2,*]

[1] Key Laboratory of Ecology and Environment in Minority Areas, Minzu University of China, National Ethnic Affairs Commission, Beijing 100081, China
[2] College of Life and Environmental Sciences, Minzu University of China, Beijing 100081, China
* Correspondence: lihua@muc.edu.cn; Tel.: +86-158-1057-5580

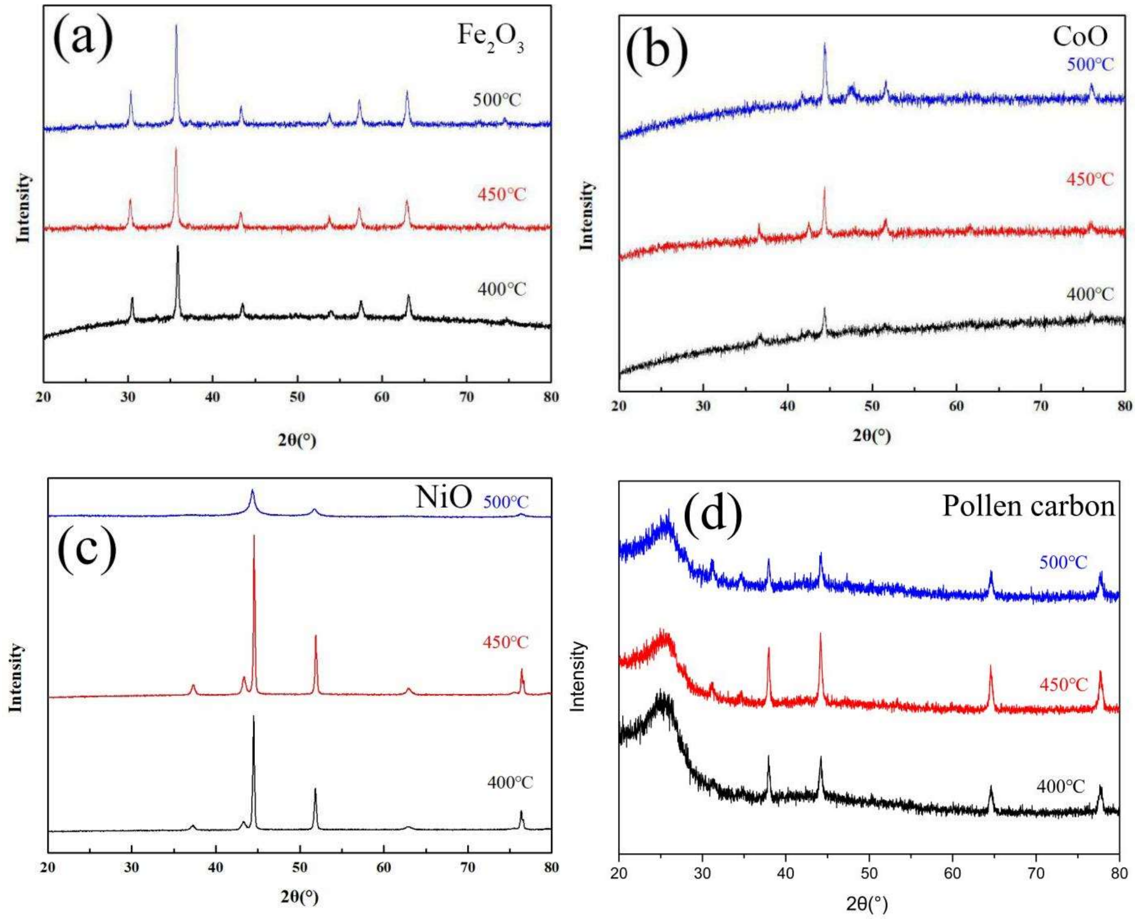

Figure S1. XRD patterns of Fe_2O_3 (a), CoO (b), NiO (c) and pollen carbon (d) at different calcination temperatures.

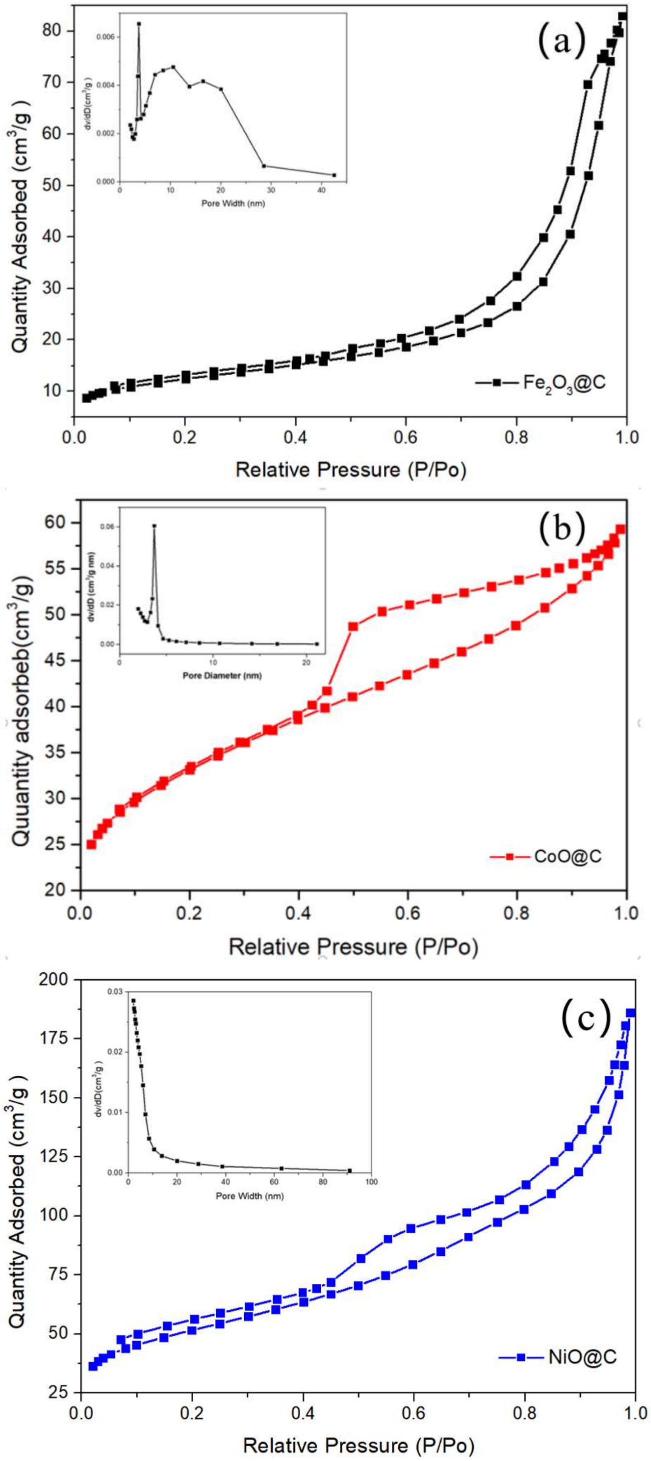

Figure S2. Adsorption isotherms and differential pore size distributions in a semi-logarithmic scale of Fe_2O_3@C-500 (a), CoO@C-500 (b) and NiO@C-500 (c).

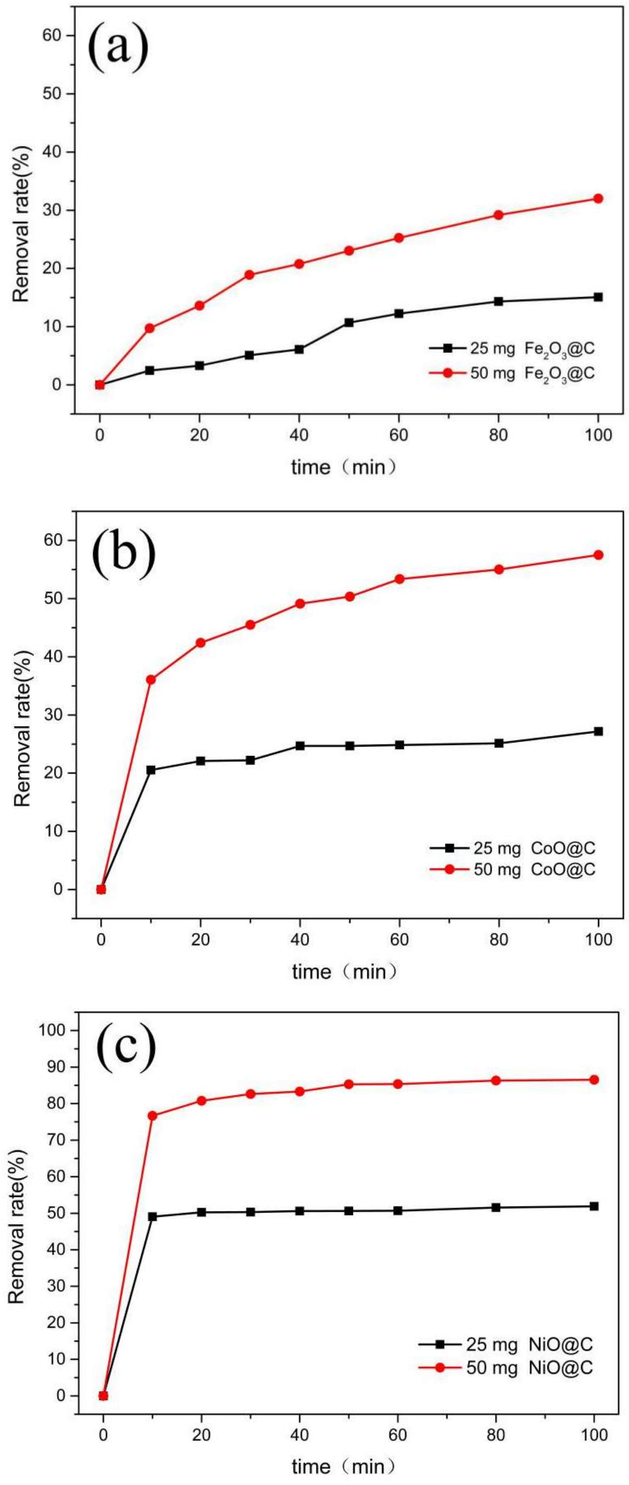

Figure S3. Adsorption degradation of Norfloxacin with 25 mg and 50 mg Fe_2O_3@C-500 (a), CoO@C-500 (b) and NiO@C-500 (c).

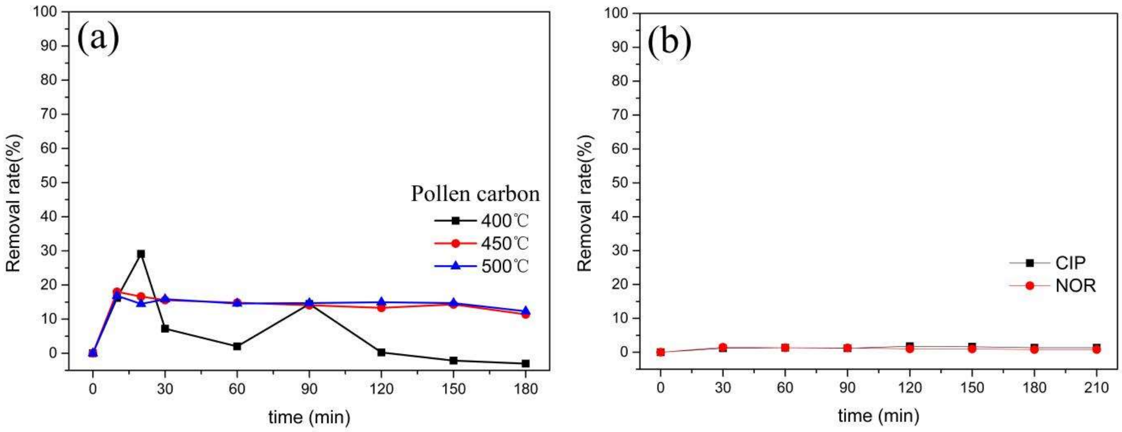

Figure S4. Removal efficiency of 20 mg/L MO (a) and CIP/NOR (b) in the adsorption process on pollen carbon(a).

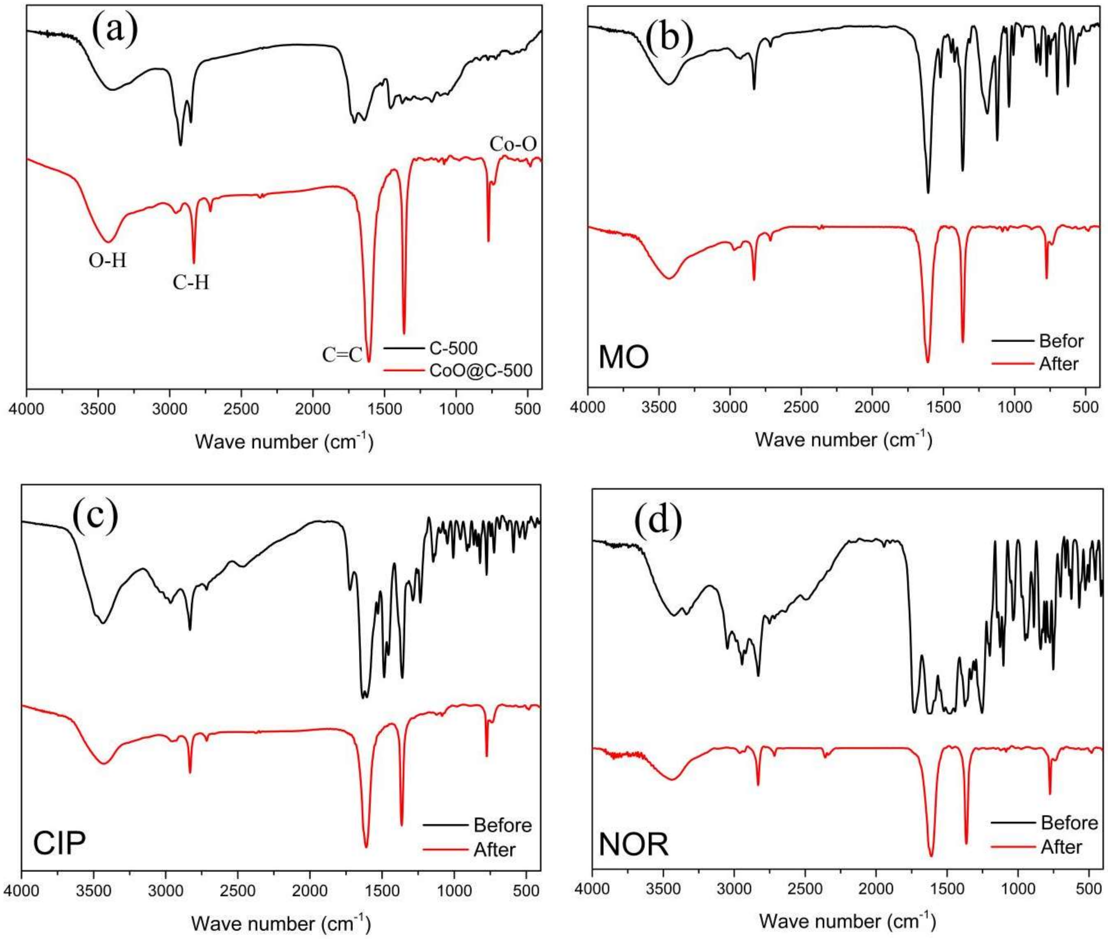

Figure S5. FT-IR of C-500 and CoO@C-500 (a), FT-IR of the MO (b), CIP (c) and NOR (d) before and after adsorption by carbon.
